# Safety and efficacy analysis of chemoradiotherapy/radiotherapy combined with nimotuzumab for treating unresectable oesophageal squamous cell carcinoma in elderly patients: a retrospective analysis

**DOI:** 10.1186/s12876-022-02602-5

**Published:** 2022-12-17

**Authors:** Yu Zhang, Jidong Wang, Di Cui, Lei Kong, Peng Wang, Zhixue Fu, Mengmeng Su, Bin Li, Jun Liang

**Affiliations:** 1 Department of Radiation Oncology, Peking University International Hospital, No.1 of Life Park Road, Life Science Park of Zhong Guancun, Changping District, Beijing, 102206 China; 2 Department of Oncology Center, Peking University International Hospital, No.1 of Life Park Road, Life Science Park of Zhong Guancun, Changping District, Beijing, 102206 China

**Keywords:** Chemoradiotherapy, Nimotuzumab, Oesophageal squamous cell carcinoma, Elderly, Treatment outcome

## Abstract

**Objective:**

To investigate the safety and efficacy of chemoradiotherapy or radiotherapy combined with nimotuzumab in the treatment of unresectable oesophageal squamous cell carcinoma (ESCC) in elderly patients.

**Methods:**

This study retrospectively analysed 54 cases of elderly patients (aged over 70 years) with unresectable ESCC in our centre between December 2016 and November 2019. The patients were treated with a radiation dose of 50–61.6 Gy (25–30 fractions) combined with nimotuzumab for targeted therapy with or without chemotherapy according to each patient’s condition. The patients were observed for quality of life, safety, side effects and survival before and after the treatment.

**Results:**

Among the 54 patients, 26 were treated with nimotuzumab combined with chemoradiotherapy and 28 were treated with nimotuzumab combined with radiotherapy. Toxicities were mainly oesophagitis (≥ Grade 2, 38.9%), myelosuppression (≥ Grade 3, 24.1%) and hypoproteinaemia (any grade, 94.4%). The rates of complete response, partial response, disease stability and disease progression were 11.1% (6/54), 81.5% (44/54), 3.7% (2/54) and 3.7% (2/54), respectively, and the overall objective response rate was 92.6% (50/54). The median follow-up time was 35.1 months, and the 1- and 2-year overall survival (OS) and progression-free survival (PFS) rates were 61.1% (1 year OS) and 35.2% (2 year OS), 42.6% (1 year PFS) and 16.7% (2 year PFS), respectively. The median OS and PFS rates were 16.0 and 10.0 months, respectively.

**Conclusion:**

Nimotuzumab combined with chemoradiotherapy or radiotherapy was well tolerated in elderly patients with unresectable ESCC. This combination can achieve a good treatment response and enhance survival.

## Introduction

Oesophageal carcinoma is one of the most common gastrointestinal cancers and ranks seventh in malignant tumours and sixth in mortality worldwide. Almost 1 in every 20 cancer deaths occurs in China [1] and China has one of the highest incidences of oesophageal carcinoma, which is the most common cause of cancer-related deaths together with lung and liver cancers in the country [2]. The primary histological type of oesophageal carcinoma in East Asia is oesophageal squamous cell carcinoma (ESCC), and despite various studies on ESCC, the treatment failure rate remains high [3].

Epidermal growth factor receptor (EGFR) is overexpressed in ESCC tissues and has been significantly associated with high local recurrence rates and low overall survival (OS) rates in multivariate analyses [4]. In recent years, it has been suggested that anti-EGFR agents may improve the outcome of oesophageal carcinoma treatment [5]. Nimotuzumab is an anti-EGFR humanised monoclonal antibody. Several in vitro studies have revealed that nimotuzumab has a radiosensitising and synergistic effect when combined with chemotherapy [6–8]. It is indicated that the toxicity of nimotuzumab is similar to that of cetuximab in combination with chemoradiotherapy, but the objective response rate (ORR) of nimotuzumab is slightly higher than that of cetuximab [9].

At present, chemoradiotherapy is the primary treatment for patients with local advanced oesophageal carcinoma [10]. For elderly patients with unresectable oesophageal carcinoma, oesophageal lesions and concomitant symptoms seriously influence patients’ nutrition, quality of life and survival; furthermore, surgery cannot be performed because of advanced age, underlying diseases or comorbidities, so it is a challenge to improve treatment outcomes in such patients. During the past decade, potential therapeutic targets for oesophageal carcinoma have been investigated, but their development has lagged behind some of other types of tumours.

A retrospective study showed that radiotherapy combined with nimotuzumab in elderly patients with oesophageal carcinoma was preliminarily proven to be safe and effective [11]. The common adverse reactions mainly included oesophagitis, pneumonia and haematologic toxicity. The incidence of Grade-3–4 adverse reactions was 17.4%, and the median OS and progression-free survival (PFS) were 17 and 10 months, respectively. However, the safety and efficacy of nimotuzumab combined with concurrent chemoradiotherapy for elderly patients with unresectable oesophageal carcinoma are not clear. Most studies on nimotuzumab combined with concurrent radiotherapy or chemotherapy did not focus on elderly patients or did not evaluate their quality of life, an outcome that may be particularly important for elderly patients with an expected short survival time.

In this study, the safety and efficacy of nimotuzumab combined with chemoradiotherapy or radiotherapy in elderly patients with ESCC were investigated.

## Materials and methods

### Patient eligibility

Elderly patients with ESCC who were treated with radiotherapy/chemoradiotherapy + nimotuzumab at our hospital from December 2016 to November 2019 were analysed retrospectively.

The inclusion criteria were as follows: (1) patients aged 70 years or older, (2) ESCC confirmed by pathology, (3) patients who were unresectable or unwilling to have surgery or endoscopic treatment, (4) Stage I–IVA (based on imaging and the American Joint Committee on Cancer’s staging [8th edition]), (5) estimated survival time ≥ 3 months, (6) Karnofsky Performance Status ≥ 70, (7) largely normal bone marrow function and (8) patients who signed an informed consent form.

Unresectable ESCC in this study was defined as follows: (1) The tumour was T4b, involving the heart, large blood vessels, trachea, vertebral body or adjacent abdominal organs, including the liver, pancreas, lung and spleen, (2) cervical or thoracic oesophageal cancer, with a lesion < 5 cm from the cricopharyngeal muscle, (3) the tumour was stage IVA at the lower oesophagus, but the regional lymph node involved blood vessels or other organs, making resection impossible or (4) unresectable because of comorbidities. Each case was discussed by a multidisciplinary team to determine whether surgery could be performed.

The exclusion criteria were as follows: (1) oesophageal adenocarcinoma or other pathological types, (2) distant metastases, (3) tumour recurrence, (4) severe infectious disease, (5) a combination of other tumours and (6) history of surgery, chemotherapy or radiotherapy.

The rejection criteria were: (1) refusal of treatment for various reasons (most of the patients who refused treatment in this study were unable to afford the cost of treatment) and (2) incomplete or missing information.

All patients in this study signed an informed consent form, and the study protocol was approved by the institutional review board of our hospital.

This study retrospectively analysed 54 elderly patients with ESCC. Among them, 28 patients received nimotuzumab combined with radiotherapy and were enrolled in an NRT group. The other 26 patients received nimotuzumab combined with chemoradiotherapy and were enrolled in an NCRT group.

### Treatment

#### Radiotherapy

All patients received intensity-modulated radiation therapy. Patients were positioned in the supine position and underwent a computed tomography (CT) scan with a 5-mm slice after the position was fixed using thermoplastic film. The target area was outlined according to the definition in the *International Commission on Radiological Units and Measurements 62*. The gross tumour volume (GTV), including the primary lesions and the involved lymph nodes, was determined according to CT or positron emission tomography (PET), endoscopy and oesophagus barium meal. Elective node irradiation was applied to the clinical target volume (CTV), which was 3 cm superior and inferior to the GTV, 1 cm horizontal to the GTV and included the corresponding lymphatic drainage areas. The planning target volume (PTV) was defined as the CTV plus 5 mm three-dimensionally. The PTV included at least 95% of the isodose lines, and 95% of the PTV dose was 50–61.6 Gy in 28–30 radiations. The radiotherapy was delivered by a Varian linear accelerator, once a day, five times a week.

The organ-at-risk dose limits were V20 ≤ 25%, V30 ≤ 20% and V5 ≤ 60% for both lungs and V40 ≤ 30% and V30 ≤ 40% for the heart. The maximum dose for the spinal cord was 45 Gy.

#### Chemotherapy

All patients received (1) tegafur–gimeracil–oteracil potassium capsules [12], 40–60 mg per dose according to the patient’s body surface area, twice a day (23/54) and (2) paclitaxel 45 mg/m^2^ d1 + cisplatin 20 mg/m^2^ d1, once a week (3/54) [13].

#### Targeted therapy

Nimotuzumab was administered at a dose of 400 mg weekly [14].

### Evaluation criteria

#### Before treatment

Eligible patients were required to complete a history interview, physical examination, blood examination, electrocardiogram, oesophagoscopy and biopsy, oesophagus barium meal, chest and abdomen CT, neck ultrasound, radionuclide bone scan and brain magnetic resonance imaging or PET/CT.

#### Evaluation of solid tumours after treatment

The evaluation of treatment efficacy was performed according to the Response Evaluation Criteria in Solid Tumours scale (version 1.1) [15], including the change in the longest diameter of target lesions (oesophageal lesions, metastatic lymph nodes) compared with baseline and the evaluation of non-target lesions.

#### Evaluation of toxicity and adverse reactions

Adverse reactions of radiotherapy (oesophagitis, pneumonia) were evaluated according to the Radiation Therapy Oncology Group’s classification of adverse reactions [16]. Haematological toxicity (including leukocyte, red blood cell and platelet reduction, low protein and low potassium) and chemotherapy adverse reactions (including nausea, vomiting and malaise) were evaluated according to the Common Terminology Criteria for Adverse Events (5.0) [17].

#### European Organisation for Research and Treatment of Cancer Quality of Life Questionnaire (QLQ-OES18) evaluation

A survey was completed by patients before and after treatment, and the scores were recorded. The scale consisted of 10 items and 18 questions (1–4 points per question) [18].

### Follow-ups

Adverse reactions were evaluated every week during the treatment. Follow-ups were conducted 1 month after the treatment, every 3 months for the subsequent 2 years, and every 6 months in years 3–5. These evaluation include each patient’s symptoms, physical examinations, routine blood tests, biochemistry results, tumour markers and imaging results; gastroscopy and biopsy were performed if necessary.

### Statistical analysis

A statistical analysis was performed using SPSS™ Statistics v26.0 software, and the Kaplan–Meier method was used for the analysis of OS and PFS. The Wilcoxon rank–sum test was used to compare the difference in QLQ-OES18 scale scores for each patient before and after treatment.

## Results

### Characteristics

A total of 54 enrolled patients were treated with nimotuzumab combined with radiotherapy or chemoradiotherapy from December 2016 to November 2019. The median age of the patients was 75 (70–91) years, and the patient information is shown in Table 1. The dose intensities of radiotherapy and chemotherapy were not adjusted in all patients.

**Table 1:** Patient information

Characteristics	Total (n = 54)	NRT group (n = 28)	NCRT group (n = 26)
Gender			
Male	42 (77.8%)	22 (78.57%)	20 (76.92%)
Female	12 (22.2%)	6 (21.43%)	6 (23.08%)
Age			
70–79 years old	41 (75.9%)	16 (57.14%)	25 (96.15%)
More than 80 years old	13 (24.1%)	12 (42.86%)	1 (3.85%)
KPS			
70–80	28 (51.9%)	19 (67.86%)	9 (34.62%)
90–100	26 (48.1%)	9 (32.14%)	17 (65.38%)
Location of the tumor			
Upper esophagus	8 (14.8%)	2 (7.14%)	6 (23.08%)
Middle esophagus	28 (51.9%)	16 (57.14%)	12 (46.15%)
Lower esophagus	18 (33.3%)	10 (35.72%)	8 (30.77%)
Stage of T			
T1	3 (5.6%)	1 (3.57%)	2 (7.69%)
T2	12 (22.2%)	5 (17.86%)	7 (26.92%)
T3	22 (40.7%)	14 (50.00%)	8 (30.77%)
T4	17 (31.5%)	8 (28.57%)	9 (34.62%)
Stage of N			
N0	16 (29.6%)	8 (28.57%)	8 (30.77%)
N1	18 (33.3%)	12 (42.86%)	6 (23.08%)
N2	19 (35.2%)	8 (28.57%)	11 (42.31%)
N3	1 (1.9%)	0 (0)	1 (3.84%)
Tumor stage			
I	3 (5.5%)	1 (3.57%)	2 (7.70%)
II	11 (20.4%)	7 (25.00%)	4 (15.38%)
III	21 (38.9%)	11 (39.29%)	10 (38.46%)
Iva	19 (35.2%)	9 (32.14%)	10 (38.46%)
Smoking	37 (68.52%)	19 (67.86%)	18 (69.23%)

NRT, nimotuzumab + radiotherapy; NCRT, nimotuzumab + chemo-radiotherapy; KPS, Karnofsky performance status; T, tumor; N, node

### Response

Patients underwent a comprehensive evaluation after treatment. Six patients (11.1%) achieved a clinical complete response, 44 patients (81.5%) achieved a partial response, 2 patients (3.7%) had disease stability, and only 2 patients (3.7%) showed disease progression. The ORR was 92.6%. The response of each group is shown in Table 2.

**Table 2:** Anti-tumor effect of both groups

	Total (n = 54)	NRT group (n = 28)	NCRT group (n = 26)
Complete response, n (%)	6 (11.1%)	2 (7.1%)	4 (15.4%)
Partial response, n (%)	44 (81.5%)	23 (82.1%)	21 (80.8%)
Stable disease, n (%)	2 (3.7%)	2 (7.1%)	0 (0)
Progressive disease, n (%)	2 (3.7%)	1 (3.6%)	1 (3.8%)
Objective response rate	92.6%	89.2%	96.2%
Median OS, months	16.0	9.0	24.0
Median PFS, months	10.0	7.0	17.0

NRT, nimotuzumab + radiotherapy; NCRT, nimotuzumab + chemo-radiotherapy; OS, overall survival; PFS, progression-free survival

### Toxicities

The treatments were well tolerated. There were no Grade-5 adverse reactions, but 17/54 (31.5%) of patients had ≥ Grade-3 adverse reactions. The five most common adverse reactions were oesophagitis, hypoproteinaemia, fatigue, leukopenia and pneumonia.

The incidence of ≥ Grade-3 haematologic reactions was 20.3% (11/54), including 2 patients with > Grade-3 leukocyte and platelet reduction. The incidence of hypoproteinaemia was 94.4% (any grade, 51/54), which has not been reported in previous studies. The incidence of ≥ Grade-2 oesophagitis was 38.9% (21/54), including 3 patients (5.6%) with Grade-3 oesophagitis. The incidence of Grade-1–2 radiation pneumonitis was 46.2% (25/54), while it was 7.4% (4/54) for Grade 3. Other adverse reactions are shown in Table 3.

**Table 3:** Acute toxicities during treatment

Item	Grade 1–2			≥ Grade 3		
	NRT (n = 28)	NCRT (n = 26)	Total (n = 54)	NRT (n = 28)	NCRT (n = 26)	Total (n = 54)
Leukopenia	19 (67.86%)	15 (57.69%)	34 (62.96%)	2 (7.14%)	6 (23.08%)	8 (14.81%)
Anemia	10 (35.71%)	13 (50.00%)	23 (42.59%)	0 (0)	0 (0)	0 (0)
Thrombocytopenia	4 (14.29%)	8 (30.77%)	12 (22.22%)	1 (3.57%)	4 (15.38%)	5 (9.26%)
Hyponatremia	5 (17.86%)	3 (11.54%)	8 (14.81%)	2 (7.14%)	2 (7.69%)	4 (7.41%)
Hypoproteinemia	27 (96.43%)	24 (92.31%)	51 (94.44%)	0 (0)	0 (0)	0 (0)
Nausea	6 (21.43%)	12 (46.15%)	18 (33.33%)	0 (0)	0 (0)	0 (0)
Fatigue	25 (89.29%)	22 (84.62%)	47 (87.04%)	0 (0)	0 (0)	0 (0)
Esophagitis	25 (89.29%)	25 (96.15%)	50 (92.59%)	2 (7.14%)	1 (3.85%)	3 (5.56%)
Pneumonia	8 (28.57%)	17 (65.38%)	25 (46.30%)	1 (3.57%)	3 (11.54%)	4 (7.41%)
Fever	2 (7.14%)	4 (15.38%)	6 (11.11%)	0 (0)	0 (0)	0 (0)

### Survival

**Fig. 1:**
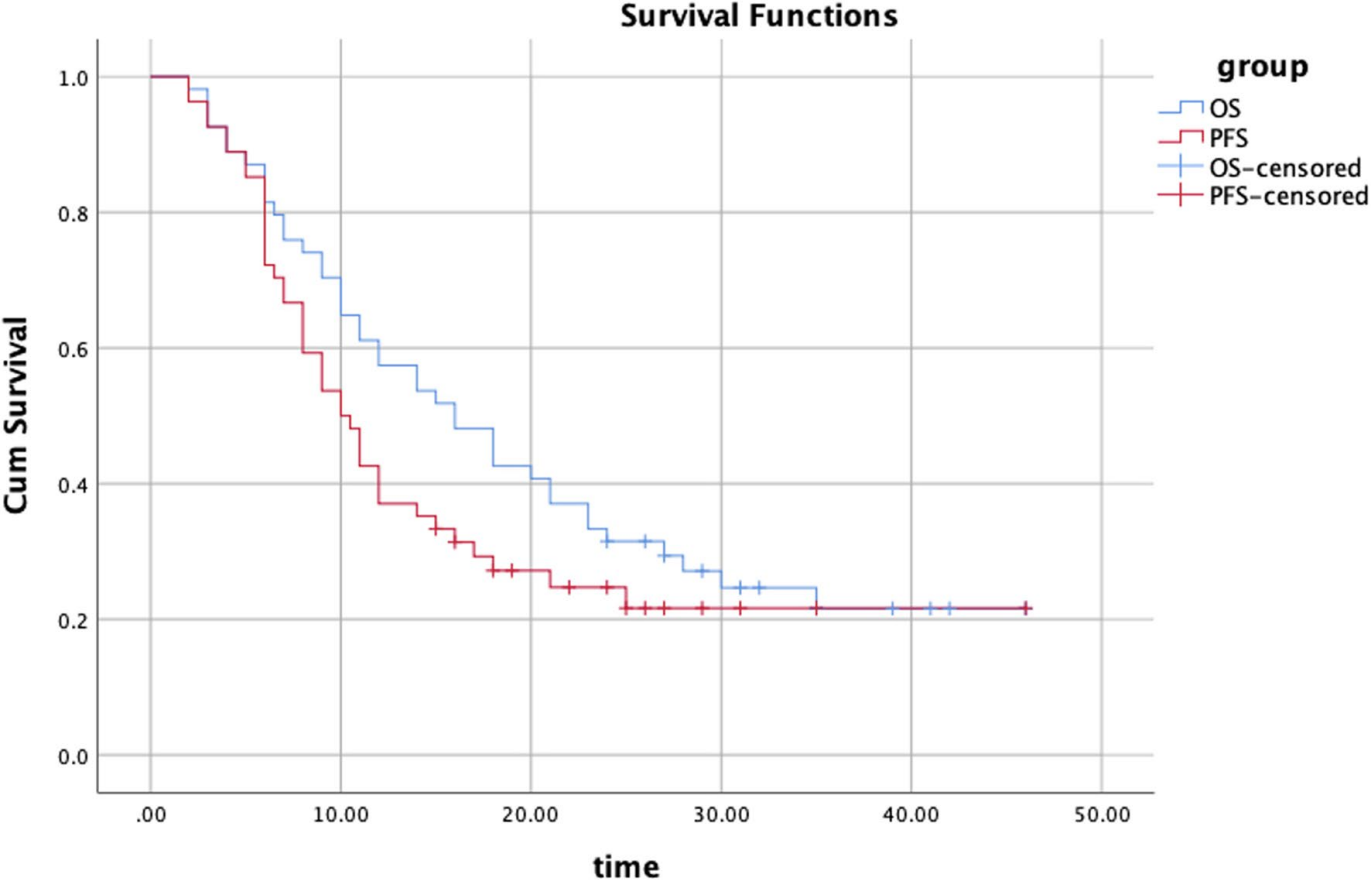
OS and PFS curves for all patients. OS, overall survival; PFS, progression free survival

The last follow-up date was 2 November 2021. The median follow-up time was 35.1 months, and 13 patients (24.1%) survived at the last follow-up, 2 of whom were treated with re-irradiation because of lymph node recurrence. Of the 41 patients who died, 3 (7.3%) died from complications (including bleeding and pneumonia), 17 (41.5%) died from other underlying diseases (including heart, kidney and lung disease), 6 (14.6%) had local and regional lymph node progression, and 15 (36.6%) had distant metastases. The median OS and PFS rates were 16.0 and 10.0 months, respectively. The 1- and 2-year OS rates were 61.1% and 35.2%, respectively, while the corresponding PFS rates were 42.6% and 16.7%, respectively (Fig. 1).

**Fig. 2:**
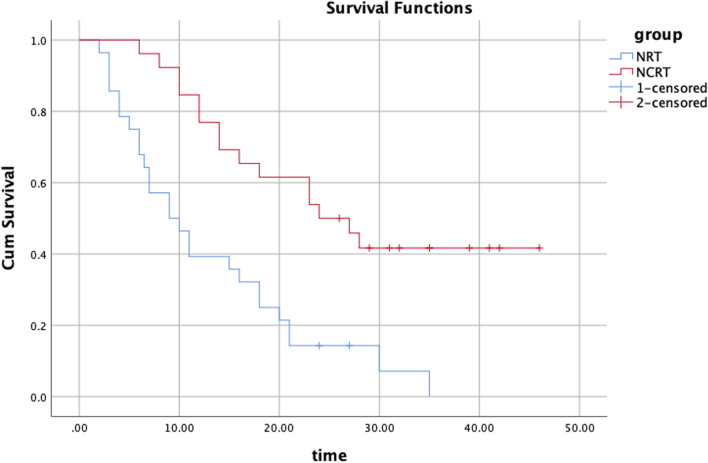
OS curves of the NRT and NCRT groups. NRT, nimotuzumab + radiotherapy; NCRT, nimotuzumab + chemo-radiotherapy; OS, overall survival

**Fig. 3:**
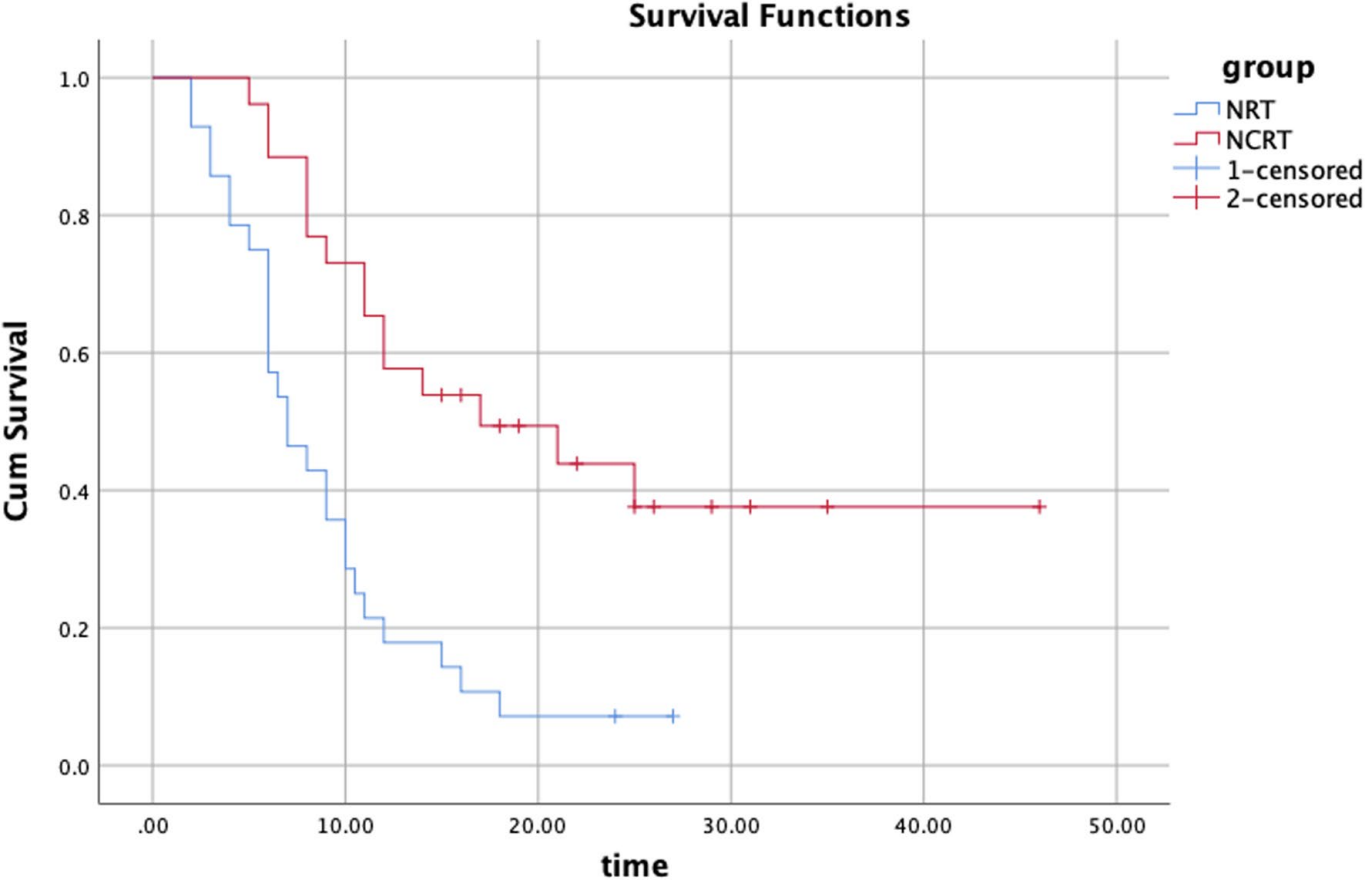
PFS curves of the NRT and NCRT groups. NRT nimotuzumab + radiotherapy; NCRT, nimotuzumab + chemo-radiotherapy; PFS, progression free survival

The median OS and PFS rates were 9.0 and 7.0 months in the NRT group and 24.0 and 17.0 months in the NCRT group, respectively. The OS and PFS rates in both groups are shown in Figs. 2 and 3, respectively.

### European Organisation for the Research and Treatment of Cancer Quality of Life Questionnaire QLQ-OES18 evaluation

The QLQ-OES18 is a scale specifically designed to evaluate the quality of life of patients with oesophageal carcinoma [19]. In our study, the questionnaire was completed by patients before and after treatment. It was found that some symptoms (dysphagia [difficulty swallowing saliva] and choking) improved after treatment (*p* < 0.05), while other symptoms (dry mouth, cough, gastroesophageal reflux and pain) worsened after therapy (*p* < 0.05). Some symptoms (diet, taste and language) did not change significantly (*p* > 0.05). The results are presented in Table 4.

**Table 4:** Comparison of QLQ-OES18 before and after treatment

	Treatment				
QLQ-OES18	Before the treatment		After the treatment		Wilcoxon rank sum *P*
Item	Mean	SD	Mean	SD	
Dysphagia	7.037	1.613	4.889	1.022	0.000
Trouble swallowing saliva	1.259	0.442	1.000	0.000	0.000
Choking	2.963	0.672	2.019	0.566	0.000
Eating	5.907	1.773	5.833	1.145	0.682
Dry mouth	1.315	0.543	1.833	0.720	0.000
Taste	1.037	0.191	1.130	0.391	0.096
Cough	1.296	0.603	1.611	0.712	0.001
Speech	1.093	0.293	1.074	0.264	0.655
Reflux	3.148	1.433	3.389	1.089	0.220
Pain	4.093	1.233	4.574	1.191	0.035

SD, standard deviation

## Discussion

Oesophageal carcinoma is usually at a late stage when it is diagnosed and is prone to metastasis, treatment resistance and frequent recurrence [20]. The clinical management of ESCC is challenging, and due to population ageing, the number of elderly patients with cancer will increase; such patients have a higher risk of death and disease progression compared with younger patients [21] and a lower tolerance to radiotherapy and chemotherapy. Previous studies have shown that elderly patients with oesophageal carcinoma may have more toxicities and more complications during treatment [22]. In this study, 24.1% of the patients (13/54) were over 80 years old, 72.2% (39/54) had varying degrees of medical diseases (including hypertension, diabetes, renal disease and pulmonary disease), and 51.9% (28/54) had Karnofsky scores of 70–80, with poor overall condition.

Elderly patients have a short life expectancy, and side effects during treatment directly influence their quality of life and survival to some extent. In this study, the QLQ-OES18 was scored before and after treatment in the enrolled patients, which was a great improvement compared with previous studies. The QLQ-OES18 is reliable, effective and acceptable for evaluating the quality of life in patients with oesophageal carcinoma [18, 23]. In this study, patients were able to cooperate in finishing the scale, and the results suggested that dry mouth, cough, gastro-oesophageal reflux and pain were worse after treatment than before (*p* < 0.05). Some changes during treatment, such as dysphagia, were obvious, but sometimes, since patients may not always let medical staff know of their discomfort, some symptoms might have gone unnoticed. These symptom changes have a great influence on the overall quality of life, which is especially important for elderly patients. Therefore, the quality-of-life scale should be more widely used in patients with oesophageal carcinoma, especially in elderly patients.

In our study, the ORR was 92.6%, the disease control rate was 96.3%, and the median OS and PFS durations were 16 and 10 months, respectively, which were similar to those reported in the NICE study in Brazil [7, 24]. The subgroup analysis showed that the NCRT group had better OS and PFS, which may have been because the patients in the NCRT group were in better condition than those in the NRT group at the beginning of the study. Patients in NCRT group seemed to have younger age and better performance status. Age and performance status are 2 factors affecting oncologists to choose the treatment protocols. Therefore, it can be speculated that elderly patients with ESCC can be actively treated with NCRT after an adequate evaluation of their age and general condition, and patients in a better general condition with relatively early staging may benefit more from NCRT. In contrast, NRT may be a better option for patients in poor general condition. While 2 of the 13 patients who survived in this study had re-irradiation for regional lymph node recurrence, they are currently surviving, with minimal adverse effects. Therefore, it can be suggested that re-irradiation is an option for elderly patients with ESCC who have regional lymph node recurrence, since it may be beneficial. In this study, 3 patients with Stage-I ESCC who were unwilling to undergo surgery were enrolled, and all achieved CR, which suggests that NCRT has few adverse effects and good efficacy for elderly patients with ESCC. Studies in larger populations are required for confirmation.

## Conclusion

In summary, nimotuzumab combined with chemoradiotherapy or radiotherapy was well tolerated in elderly patients with unresectable ESCC. This combination can achieve a good treatment response and enhance survival. The sample size of our study was relatively small because of the number of elderly patients admitted to the single centre. In addition, it was a retrospective study without a control group, so the results have limitations and need to be investigated further through large-sample prospective studies.

## Data Availability

All data generated or analyzed during this study are included in this published article.

## Abbreviations

OS: Overall survival
PFS: Progression-free survival
ESCC: Oesophageal squamous cell carcinoma
EGFR: Epidermal growth factor receptor
ORR: Objective response rate
CT: Computed tomography
GTV: Gross tumour volume
CTV: Clinical target volume
PTV: Planning target volume
